# Differential scanning fluorimetry to assess PFAS binding to bovine serum albumin protein

**DOI:** 10.1038/s41598-024-57140-9

**Published:** 2024-03-18

**Authors:** Jessica Alesio, Geoffrey D. Bothun

**Affiliations:** Department of Chemical Engineering, University of Rhode Island, Kingston, RI 02881 USA

**Keywords:** Environmental sciences, Chemistry

## Abstract

The rapid screening of protein binding affinity for poly- and perfluoroalkyl substances (PFAS) benefits risk assessment and fate and transport modelling. PFAS are known to bioaccumulate in livestock through contaminated food and water. One excretion pathway is through milk, which may be facilitated by binding to milk proteins such as bovine serum albumin (BSA). We report a label-free differential scanning fluorimetry approach to determine PFAS–BSA binding over a broad temperature range. This method utilizes the tryptophan residue within the protein binding pocket as an intrinsic fluorophore, eliminating the need for fluorophore labels that may influence binding. BSA association constants were determined by (a) an equilibrium-based model at the melting temperature of BSA and (b) the Hill adsorption model to account for temperature dependent binding and binding cooperativity. Differences in binding between PFAS and fatty acid analogs revealed that a combination of size and hydrophobicity drives PFAS binding.

## Introduction

Poly- and perfluoroalkyl substances (PFAS) are synthetic compounds that have been used in a wide variety of applications, including textile production, waterproofing, and firefighting. Once released into the environment, they are resistant to degradation due to the strength of the carbon–fluorine bond. This stability allows PFAS to enter soil, vegetation, and drinking water of livestock such as cattle^1,2^, which are exposed to PFAS through contaminated feed and water^3^. At environmentally relevant concentrations in short term studies, PFAS have not been shown to cause detrimental health effects in cattle, but additional studies are ongoing to investigate long term health effects^3,4^. The half-lives of perfluorooctanoic acid (PFOA) and perfluoroctanesulfonic acid (PFOS) in cattle range from 19 h to 1.3 days and 39–120 days, respectively and vary by sex^4–6^. The higher values for PFOS half-lives reflect the nature of PFOS and higher chain length PFAS to partition into protein-rich compartments such as the liver and blood^7^. The half-lives of other PFAS have not yet been experimentally determined for cattle. PFAS are known to be excreted through cow’s milk as well as stored in muscle tissue^6,8,9^. The milk excretion pathway poses a substantial problem for dairy farmers, as PFAS have been detected in raw and processed samples^10^.

Although PFAS transfer into milk and storage in tissue has been documented, the process by which this transport occurs is poorly understood. One predictive tool that can be used is binding affinity to bovine serum albumin (BSA), as this protein is a major component in cow blood, is stored in muscle tissue, and transfers into cow milk^11^. PFAS are known to bind to proteins and travel through the bloodstream, one suspected reason for their long half lives in cattle^3,12^. Van Asselt et al.^9^ proposed a pharmacokinetic model in which the PFAS bound to protein are cleared via excretion into cow’s milk. It should be noted that direct links of PFAS transfer by BSA to bovine milk have not been reported, however, given that BSA-PFAS binding affinity has been reported, this mechanism should be considered along with the reported mechanisms of PFAS transfer by fats and lipids. Thus, an understanding of relative protein binding of different PFAS species is essential to predicting relative PFAS amounts in milk.

In the context of PFAS-protein binding, BSA has been used primarily as a model protein with 76% homology to human serum albumin^13^. Experiments are often performed at constant temperatures between room temperature and physiological temperature, or approximately 20 °C to 37 °C. Expanded temperature ranges have been examined to extract the enthalpy and entropy of protein-PFAS binding based on the temperature-dependence, but these do not typically approach temperatures associated with protein denaturing or milk pasteurization processes. Temperatures for cow’s milk pasteurization range from 63 to 72 °C depending on the duration that the milk is held at temperature^14^. This presents an opportunity to reduce the amount of PFAS bound to protein as both elevated temperatures and denaturing dramatically reduce the binding affinity by reducing the strength of the intermolecular binding interactions and by disrupting the tertiary protein structure that form the binding domains^15,16^.

In addition, rapid screening tools to assess PFAS-protein binding are sorely needed. The most common methods used to probe this interaction are fluorescence spectroscopy, equilibrium dialysis, and fluorine nuclear magnetic resonance spectroscopy^17–23^. Although these techniques are widely used, they are also time consuming. While there are over 4,000 different compounds in the PFAS class, rapid and high throughput methods are essential for screening.

Recently, Differential Scanning Fluorimetry (DSF) has been proposed as a rapid, high throughput screening technique to quantify protein binding of various ligands, including drugs and small molecules^24,25^. DSF works by monitoring a change in fluorescence emission as a function of temperature. The most common method involves the use of a hydrophobic dye or “label” that fluoresces when bound to unfolded (denatured) protein^26^. Jackson et al. used DSF to investigate the binding of a wide range of legacy and emerging PFAS to human serum albumin (HSA) using the dye GloMelt™ as an indicator.^27^ For BSA, a label free option exists, as the tryptophan amino acid residue in the binding site is itself fluorescent and thus can be used as the fluorophore^28^. This technique reduces the potential for confounding effects from extrinsic fluorophores that may interfere with ligand binding or influence protein unfolding^29^. Based on molecular docking studies, PFOA and PFOS bind to BSA near tryptophan residue TRP-237, which was reported to be in close proximity to both PFAS bound at Sudlow site I (PFOA)^17,30^ and site II (PFOS)^17^. As this binding event occurs, the proximity of the ligand to the fluorophore induces fluorescence quenching, which can be monitored by the change in fluorescence emission. This principle has previously been used by our group and others in fluorescence spectroscopy-based investigations of PFAS binding to BSA^21^.

In this work, PFAS binding to BSA was examined using DSF from 35 to 95 °C to inform future approaches to PFAS remediation from bovine milk, particularly those that would benefit from reduced protein binding affinity at elevated temperatures. The PFAS examined include common perfluorocarboxylic acids (PFCAs; PFOA, perfluorononanoic acid (PFNA), perfluorodecanoic acid (PFDA), and perfluoro-2-methyl-3-oxahexanoic acid (GenX)) and perfluorosulfonic acids (PFSAs; perfluorobutanesulfonic acid (PFBS), perfluorohexanesulfonic acid (PFHxS), and PFOS), as well as three fatty acids analogs (octanoic acid (OA), nonanoic acid (NA), and decanoic acid (DA)) that have the same carbon chain length as the PFCA (Table 1). Warfarin, a drug compound known to bind strongly to serum albumin protein at Sudlow site I^33^, was used as a control. The label-free approach was used to determine the effect of PFAS binding on the protein denaturing or melting temperature (T_m_) and the PFAS-BSA association constant, K_a_, based on the Hill adsorption model. This model has proven more versatile than the classic Stern–Volmer quenching model and provides additional insight into protein-PFAS binding, such as binding cooperativity^21,34^.

**Table 1:** Compounds examined with reported octanol/water partition coefficients (*Log K*_*ow*_).

Compound	Molecular Formula	CompTox^a^ identifier	Log K_ow_^b^
Perfluorooctanoic acid (PFOA)	C_8_HF_15_O_2_	DTXSID8031865	5.68 (3.10)^c^
Perfluorononanoic acid (PFNA)	C_9_HF_17_O_2_	DTXSID8031863	6.51 (3.54)^c^
Perfluorodecanoic acid (PFDA)	C_10_HF_19_O_2_	DTXSID30318602	7.32 (4.15)^c^
Perfluoro-2-methyl-3-oxahexanoic acid (GenX)	C_6_HF_11_O_3_	DTXSID70880215	5.41
Sodium perfluorobutylsulfonate (PFBS)	C_4_F_9_NaO_3_S	DTXSID10896633	2.20
Sodium perfluorohexanesulfonate (PFHxS)	C_6_F_13_NaO_3_S	DTXSID90892476	2.91
Sodium perfluorooctanesulfonate (PFOS)	C_8_F_17_NaO_3_S	DTXSID50635462	4.94
Octanoic acid (OA)	C_8_H_16_O_2_	DTXSID3021645	2.99 (3.05)^c^
Nonanoic acid (NA)	C_9_H_18_O_2_	DTXSID3021641	3.41 (3.42)^c^
Decanoic acid (DA)	C_10_H_20_O_2_	DTXSID9021554	4.03 (4.09)^c^
Warfarin	C_19_H_16_O_4_	DTXSID5023742	2.86 (2.70)^c^

^a^EPA CompTox Chemicals Dashboard^31,32^.

^b^Predicted average.

^c^Experimental average.

## Results and discussion

**Figure 1:**
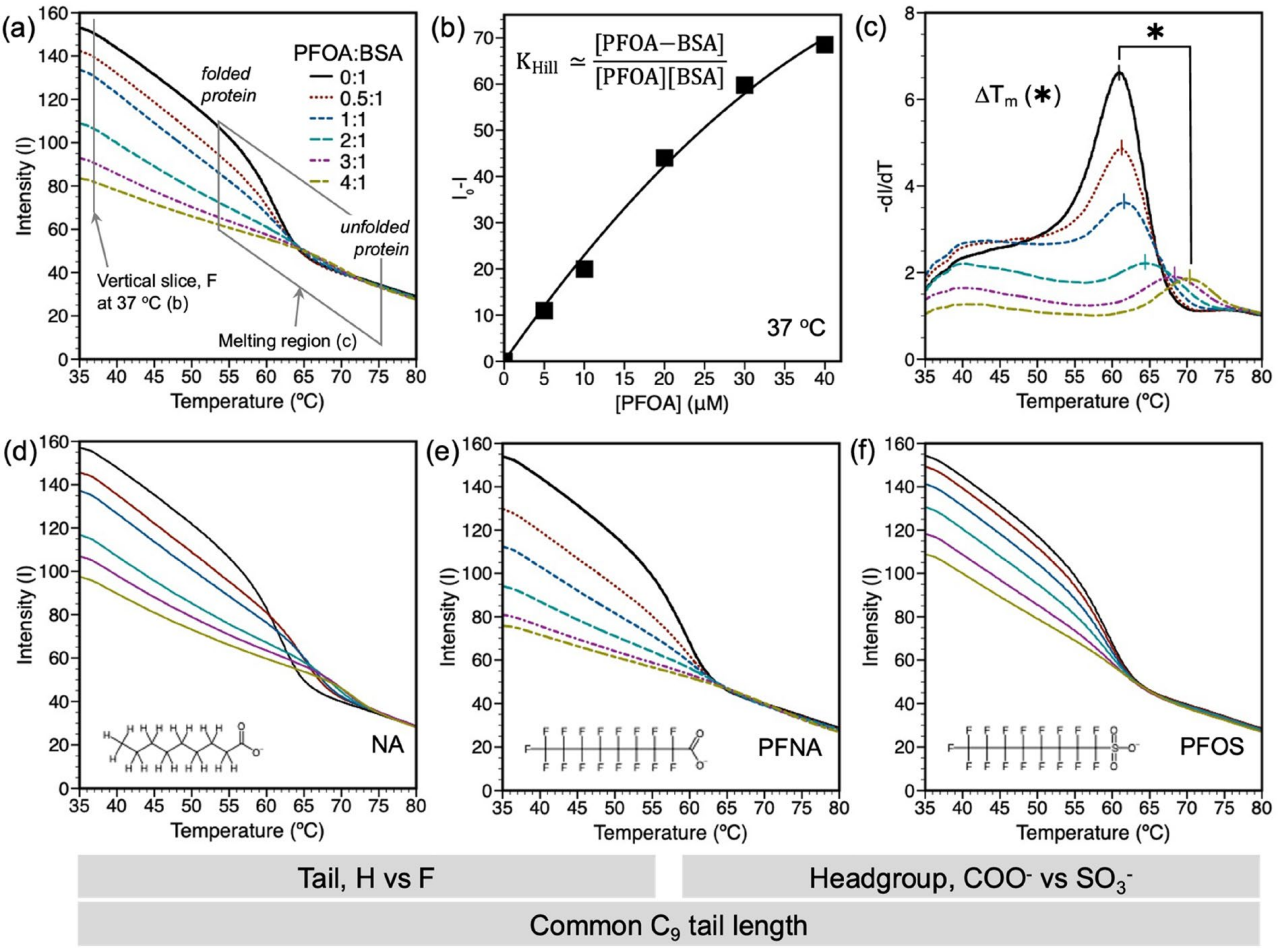
Fluorescence intensity (I) from 35 to 80 °C depicting ligand quenching of BSA. The BSA concentration in pH 7.4 PBS buffer was constant at 10 μM. PFAS:BSA ratios ranged from 0:1 to 4:1. Panes (**a**-**c**) are specific to PFOA and demonstrate the vertical transit at 37 °C from which a (**b**) Hill model plot can be created to calculate K_Hill_ and n_Hill_, and (**c**) the folded to unfolding transition from which the protein melting temperature, T_m_, can be determined. Panes (**d**-**f**) are the quenching curves for NA, PFNA, and PFOS, respectively, and compare the effects of headgroup and alkyl or fluoroalkyl tails for a common number of tail carbons.

Results from DSF are demonstrated in Fig. 1. Figure 1a depicts the analysis approach where vertical transects were used to determine K_a_ (approximated as K_Hill_, Fig. 1b) at a given temperature based on fluorescence quenching and the melting region associated with BSA unfolding was used to determine K_a_, which is inversely proportional to the dissociation constant K_d_, as well as the BSA melting temperature, T_m_ (Fig. 1c). PFAS or FA binding did not shift the maximum fluorescence emission wavelength, indicating that fluorescence intensity was not influenced by ligand concentration for a given temperature. All the compounds studied resulted in fluorescence quenching, indicating that they all bind to BSA and lead to changes in tryptophan fluorescence. This is depicted in Fig. 1d-f for NA, PFNA, and PFOS; compounds with the same number of carbons, but with different headgroups (carboxylic acids NA, PFNA; sulfonic acid PFOS) and alkyl (NA) or fluoroalkyl (PFNA, PFOS) tails.

### Quantitative analysis of binding constants at T_m_

We first determined the equilibrium-based binding constant evaluated near the melting temperature of pure BSA from the DSF curves (e.g., Fig. 1d-f). This method has been reported to yield a more accurate binding constant because it relies upon a simple model of coupled equilibrium states for both the protein and the ligand that accounts for protein folding and ligand binding^29^. The underlying assumption is that a ligand binds to the folded protein and not to the unfolded protein. Table 2 summarizes the association constants calculated at a temperature of 61 ºC. This temperature were selected because there were measurable differences between the fraction of unfolded protein for all of the PFAS and fatty acid complexes over the range of ligand:protein molar ratios, which is essential to applying Eqs. (1) and (2) to determine K_d_ = K_a_^−1^ (see “Materials and methods” section). Differences in the faction of unfolded protein, ${f}_{U}$, for PFNA are shown in Figure S1 as a function of temperature for the different PFNA:BSA ratios. In this example, if a temperature below 61 °C was selected it would precede BSA melting at the highest PFNA:BSA ratio (${f}_{U}$ ≈ 0) and if a temperature above 63 °C was selected it would exceed BSA melting and the lowest PFNA:BSA ratio (${f}_{U}$ ≈ 1). Warfarin was used as a positive control and its K_a_ agrees with values reported in the literature.^35^ It should be noted that K_a_ for PFNA and PFHxS exceed that of warfarin, which was designed as a pharmaceutical drug to bind to BSA.

**Table 2:** Association constants at 61 °C obtained by fitting to Eqs. (1) and (2).

Compound	*K*_*a*_ (10^4^ M^−1^)^a^
PFOA	2.44 (0.40)
PFNA	96.6 (52.8)
PFDA	0.33 (0.26)
GenX	1.98 (0.41)
PFBS	2.08 (0.61)
PFHxS	9.40 (3.30)
PFOS	2.73 (1.38)
OA	0.77 (0.06)
NA	293 (182)
DA	3.62 (1.11)
Warfarin (Control)	9.37 (2.24)

^a^Standard error shown in parentheses.

**Figure 2:**
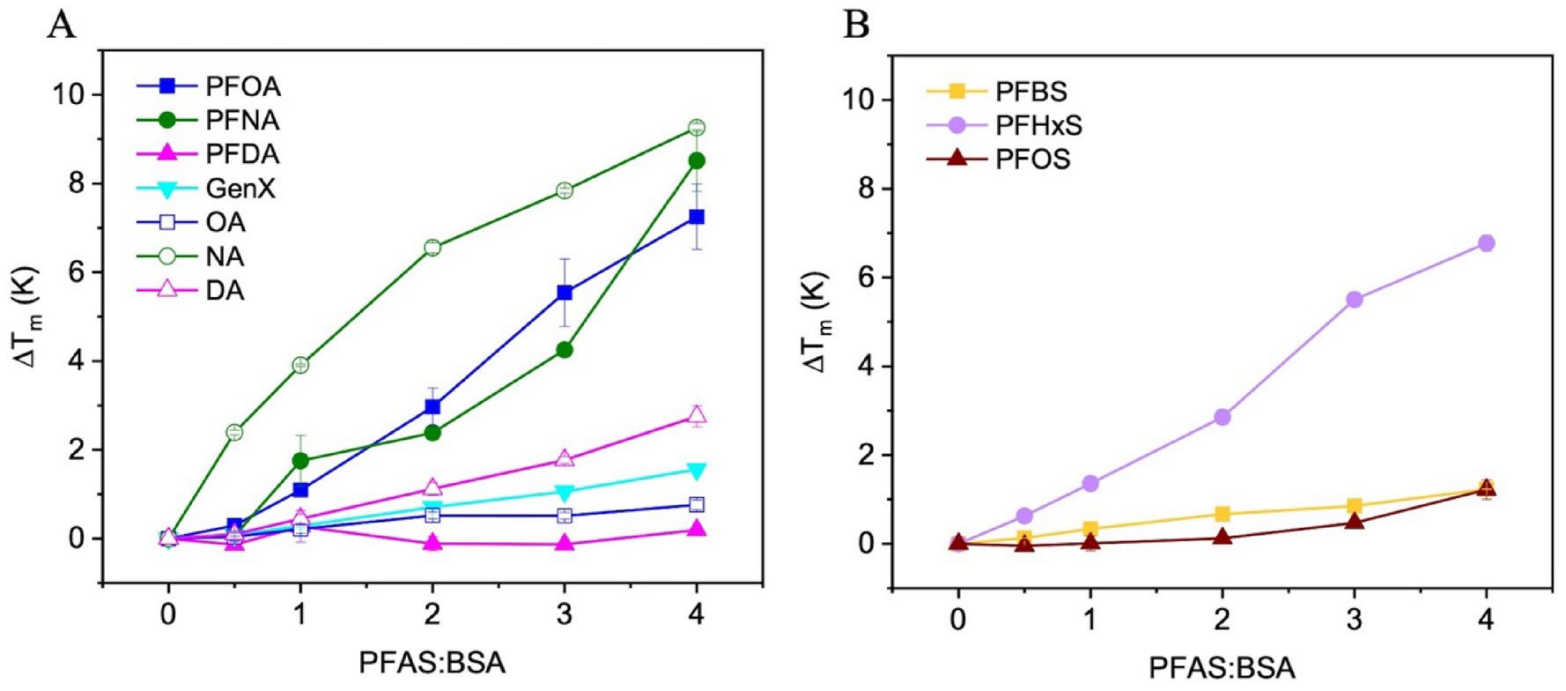
The change in melting temperature upon addition of PFAS or fatty acid to BSA as a function of the ligand:protein ratio. Filled symbols indicate PFAS compounds and open symbols indicate fatty acids. Carboxylic acids are shown in (**A**) and sulfonic acids in (**B**). Each experiment was performed in triplicate and error bars not visible are smaller than the symbols.

We then examined the stabilizing effect of PFAS or FA on BSA structure based on increases in the protein melting temperature, ΔT_m_^36,37^. All PFAS compounds stabilized the folded structure of BSA, requiring greater thermal energy for protein melting with increasing PFAS concentration (Fig. 2). Oleic and nonanoic acids also stabilized the folded protein. There was a positive qualitative relationship between compounds that stabilized the folded protein and K_a_. PFNA, PFHxS, and NA had the greatest stabilizing effect and the highest K_a_ values for their chemical group, while GenX and PFBS led to little stabilization with low K_a_ values. The most effective stabilizing PFAS were PFOA, PFNA, and PFHxS, resulting in T_m_ values ranging from roughly 69 to 71 °C whereas the native protein T_m_ is 62 °C. Temperatures exceeding 69 °C to 71 °C would be required to desorb PFAS from BSA (e.g., during pasteurization).

**Figure 3:**
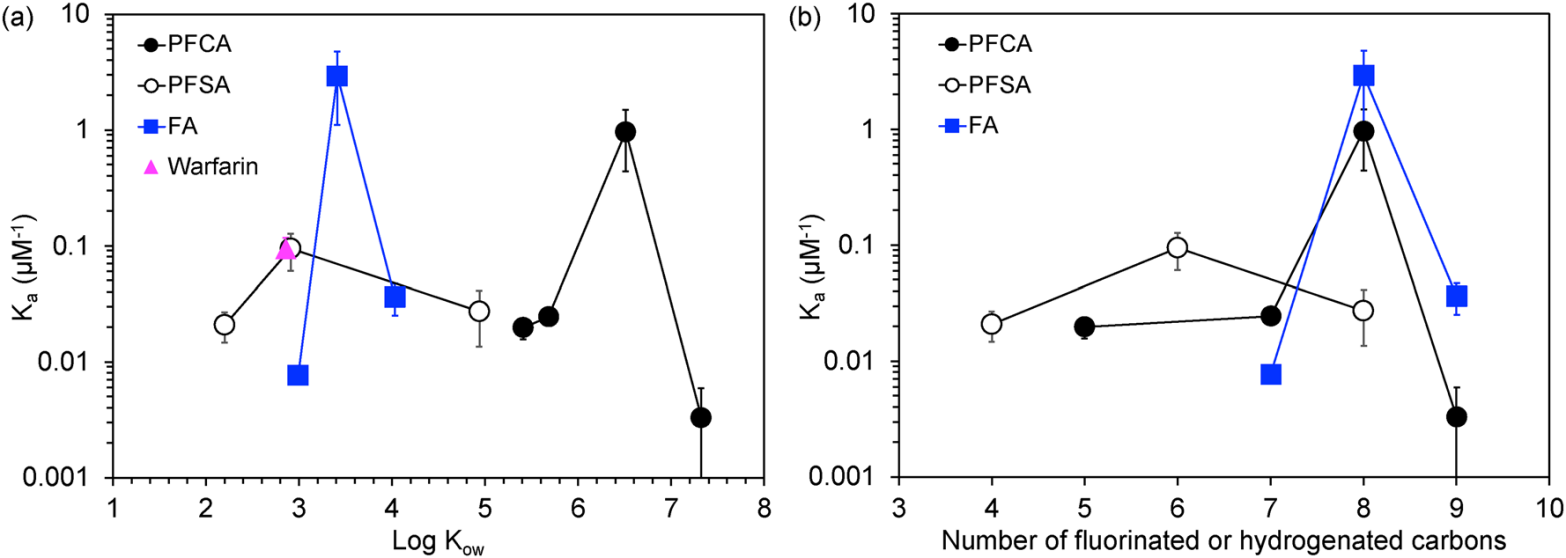
PFAS-BSA association constants (K_a_) for PFAS and fatty acids determined near the observed melting temperature of BSA (61 °C) based on the equilibrium-based binding model plotted against (**a**) Log Kow and (**b**) the number of fluorinated or hydrogenated carbon atoms in the PFAS or FA.

Ligand binding is determined by the intermolecular interactions with the protein binding pocket and the size of the ligand. A compound may be very hydrophobic (high Log K_ow_), which would lead to strong interactions within the hydrophobic binding pocket but may exhibit low binding if it is large and sterically hindered from fitting within the pocket. This is observed for PFDA, which has the highest Log K_ow_ of the PFAS examined but showed the lowest K_a_ with no measurable protein stabilization. K_a_ values were plotted a function of Log K_ow_ (predicted values, Table 1; EPA CompTox Chemicals Dashboard^31,32^) and the number of fluorinated or hydrogenated carbons (Fig. 3). An inverted “V” shaped trend is observed for all compound classes (PFCA, PFSA, FA) with PFNA, PFHxS, and NA exhibiting the greatest binding affinity, respectively. Similar trends, often described as an inverted “U” have been reported for PFAS-BSA binding behavior^27^, with, for example, PFHxS exhibiting the highest K_a_ and PFDA the lowest K_a_ for the compounds plotted. The trend substantiates previous results that report that PFAS chain lengths greater than C_8_ (i.e., greater than PFNA or PFOS) do not “fit” as well into the BSA binding pocket compared to shorter PFAS. Octanoic acid and decanoic acid both have smaller values of Log K_ow_ than PFOA and PFDA and the same carbon chain length, respectively. When comparing PFOA and OA, the higher hydrophobicity likely drives the higher association constant for PFOA. On the other hand, PFDA is larger than DA arising from its fluorinated carbons and its size hinders its binding to BSA.

PFAS that bind strongly to BSA have been detected in cattle muscle and cow’s milk, suggesting that protein binding is a driver of PFAS transport and excretion. Kowalczyk et al.^8^ determined the percent of an ingested PFAS dose that is excreted in cow’s milk and stored in muscle for PFOS, PFHxS, PFOA, and PFBS. Their study indicated that PFOS transfers more efficiently into cow’s milk than PFOA or PFBS, which is consistent with PFOS having a higher K_a_ (Table 2).

### Binding cooperativity as evidenced by the Hill adsorption model

Neither the equilibrium-based model nor the Stern–Volmer model account for (1) the fluorescence of the protein–ligand complex and (2) binding cooperativity. These models assume a 1:1 binding event, which is not necessarily the case for PFAS-BSA but is a convenient simplification. At high concentrations of PFAS relative to BSA, more than one PFAS may bind to the protein. Thus, the Hill adsorption model was used to reflect binding cooperativity over the range of temperatures studied. For this analysis only PFAS are considered. The ability to determine binding constants and cooperativity for a range of temperatures, as well as equilibrium-based binding near T_m_ and protein stabilization resulting in increases in T_m_, from a single PFAS data set demonstrates the power of the DSF technique.

**Figure 4:**
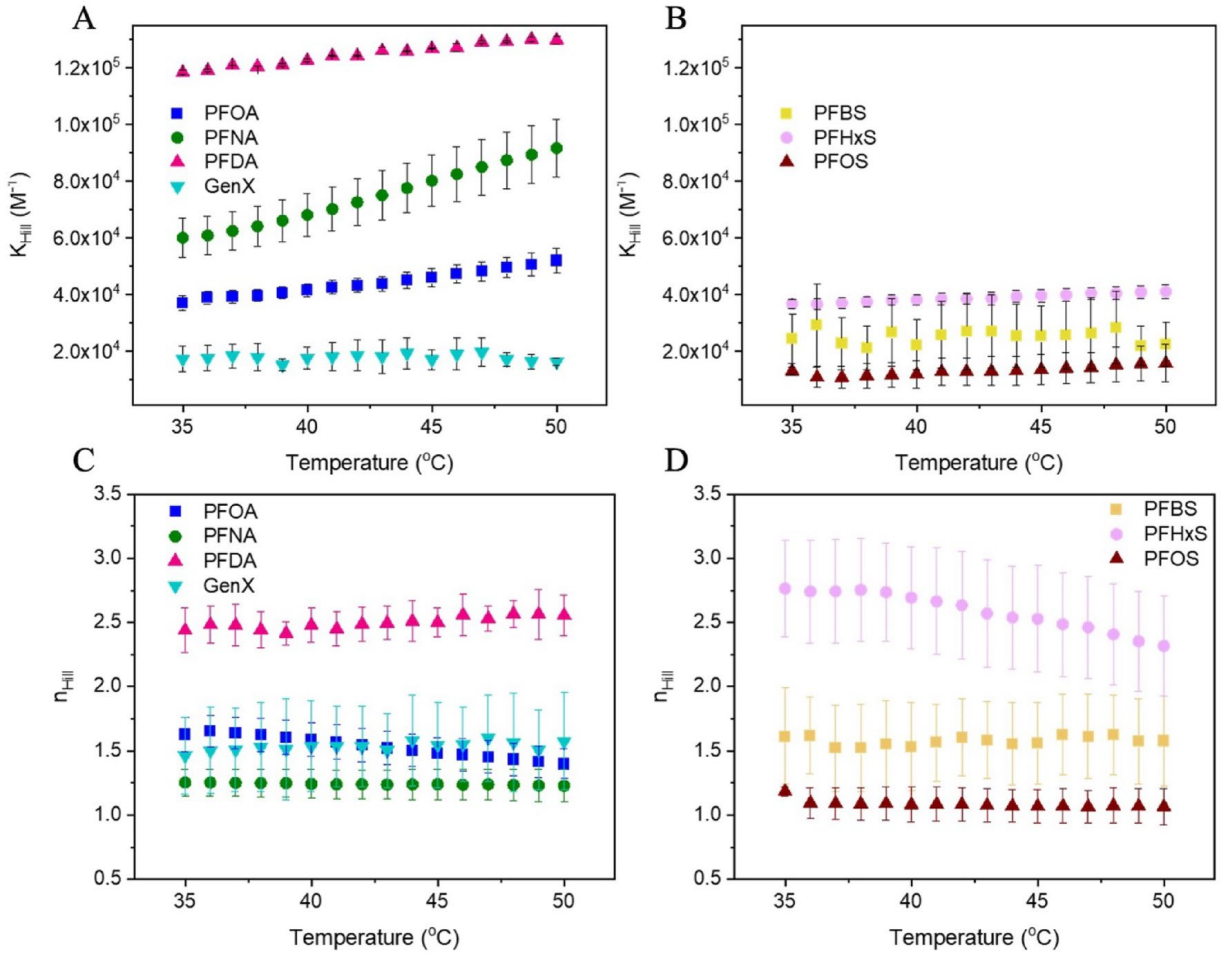
The Hill association (K_Hill_;** A**,** B**) and cooperativity (n_Hill_;** C**,** D**) constants spanning 35 °C to 50 °C modeled by Eq. 4. PFCA are shown in (**A**, **C**) and PFSA in (**B**, **D**). Each experiment was performed in triplicate and error bars not shown are smaller than the symbols.

The association constant obtained via the Hill model (Fig. 4) demonstrates the dependence of binding on temperature. An increase in K_Hill_ with temperature reflects a binding process where the entropy gain associated with water desolvation of the PFAS is greater than the reduction of enthalpic interactions^18^. This is observed for all PFCAs except for GenX, where the entropic and enthalpic changes are balanced and K_Hill_ does not change with temperature (Fig. 4A,B). PFOA, PFNA, and PFDA show the largest entropy gains based on K_Hill_ increasing with temperature, consistent with their longer tails requiring a greater water solvation cavity (and greater entropy when that cavity is no longer required due to protein binding). For PFASs, protein binding based on K_Hill_ did not exhibit temperature dependence. PFOS might be expected to show a similar trend to PFNA given its C_8_ fluoroalkyl tail, but the low K_Hill_ is consistent with the low K_a_ (Table 2) and minimal shift T_m_ (Fig. 2B). Differences in the temperature dependence of PFNA and PFOS could be due to many factors intrinsic to the PFAS (e.g., headgroup), to the protein (e.g., binding site location), and the subsequent intermolecular interactions.

Considering binding cooperativity (Fig. 4C,D), when n_Hill_ is less than 1 the binding of one ligand negatively impacts the binding of additional ligands. When n_Hill_ is equal to 1, there is no effect and when n_Hill_ is greater than 1, cooperativity is positive and the binding of one ligand positively influences the binding of additional ligands. For all PFAS n_Hill_ is greater than one, indicating positive binding cooperativity. This result provides valuable insight, especially into the behavior of PFHxS, when combined with the other analyses. PFHxS showed a higher value of K_a_ and K_Hill_ than PFOS in the equilibrium-based model despite being less hydrophobic. The high degree of binding cooperativity may account for the mechanism driving these seemingly disparate results. When PFHxS binds it cooperatively increases binding of additional ligands, leading to a larger association constant. PFDA is another interesting case; its K_a_ was low due to steric hinderances within the binding site but K_Hill_ and n_Hill_ were the largest of the PFCAs. This may reflect BSA quenching caused by non-specific PFDA adsorption outside of the binding pocket. Using fluorine nuclear magnetic resonance spectroscopy, we have previously reported that PFAS can adsorb non-specifically and that long PFAS (PFNA) may form hemimicelles on the protein surface. PFDA would be expected to exhibit even greater adsorption, perhaps enhanced by high binding cooperativity.

This work presents a new, rapid approach to analyze DSF data to aid in the rapid measurement of PFAS binding to BSA. By combining the equilibrium-based model at the melting temperature and the Hill adsorption model, valuable insight can be gained in terms of the binding mechanisms. While the equilibrium-based model offers a way to determine binding based on protein melting, the Hill adsorption model can provide binding and cooperativity constants over a range of temperatures. The understanding of temperature-based binding constants can aid in PFAS remediation and could inform pasteurization processes. In this case temperatures well beyond the melting temperature of PFAS:BSA complexes would be needed to desorb PFAS. The kinetics of BSA denaturing must also be consider. Times of half-conversion (folded to unfolded BSA) of 13.6 min and 1.5 min have been reported at 65 °C and 80 °C, respectively^38^. As specified by the USDA^39^, vat pasteurization at 63 °C for 30 min or high heat pasteurization at 88 °C for 1 s would partially desorb PFAS depending on the PFAS species and how it stabilized the protein and increased the melting temperature, but could be insufficient for significantly reducing the amount of bound PFAS.

## Materials and methods

### Material

Bovine serum albumin (lyophilized powder, 99% fatty acid free) was obtained from Sigma-Aldrich (St. Louis, MO). A BSA concentration of 10 μM in pH 7.4 phosphate buffered saline (PBS) was used in each experiment. The solution of BSA was kept at 4 °C prior to use.

Perfluorooctanoic acid (PFOA), perfluorononanoic acid (PFNA), perfluorodecanoic acid (PFDA), perfluoro-2-methyl-3-oxahexanoic acid (GenX), perfluorooctanesulfonic acid (PFOS), perfluorohexanesulfonic acid (PFHxS), and perfluorobutanesulfonic acid (PFBS) were obtained from Accustandard, Inc (New Haven, CT). Octanoic acid (OA), nonanoic acid (NA), and decanoic acid (DA) were obtained from Fisher Scientific (Waltham, MA) and warfarin was obtained from Millipore Sigma. PFAS and fatty acid solutions in PBS were prepared at least a day in advance and stored at room temperature in polypropylene containers. PFAS and FA stock solutions were prepared at 1 mM, which is below reported critical micelle concentrations for the compounds examined^40–43^. The molecular formula, EPA CompTox identifier, and reported octanol/water partition coefficients (Log K_ow_) are summarized in Table 1^31,32^.

### Differential scanning fluorimetry

Experiments were conducted using a Tycho NT.6 differential scanning fluorimeter (NanoTemper, Munich, Germany). PFAS was added to 2 mL of the stock BSA solution to obtain the PFAS:BSA or FA:BSA ratios of 0:1, 0.5:1, 1:1, 2:1, 3:1, and 4:1. Solutions were prepared a day in advance of the experiment and stored at 4 °C. Immediately prior to analysis, each sample was loaded into a 1 mm diameter glass capillary and placed in the instrument. Thermal scans were conducted from 35 to 95 °C at a scan rate of 0.5 °C s^−1^ with a 285 nm excitation wavelength. Fluorescence intensity was measured at 0.1 °C intervals at emission wavelengths of 330 and 350 nm. The ratio of the intensity at these wavelengths, I_350_/I_330_, was used to account for blue shifts in the emission peak wavelengths with increasing temperature. The minimum value of the first derivative of the I_350_/I_330_ curve with respect to temperature, denoting the midpoint of the melting transition where the fraction of folded to unfolded protein is 1, was taken as the melting temperature T_m_. Each experiment was performed in triplicate. The same procedure was used for the control ligand warfarin.

### Melting point association constant

As described by Eftink and Ghiron^44^, baseline subtraction of the raw fluorescence intensity data collected at 350 nm was performed at both high and low temperatures. The plots were then normalized by dividing by the maximum intensity value. These data were fit to Eqs. (1) and (2) to obtain a value for K_d_ which is the inverse of the association constant (K_d_ = K_a_^−1^)^29^.

1 $${f}_{U}=\frac{[U]}{\left[U\right]+\left[F\right]+[FL]}=\frac{1}{1+\frac{1}{{K}_{U}}\left(1+\frac{\left[L\right]}{{K}_{d}}\right)}$$

2 $$\left[L\right]=\frac{1}{2}\left[\left({\left[L\right]}_{t}-{\left[P\right]}_{t}-{K}_{d}\left(1+{K}_{U}\right)\right)+\sqrt{{\left({\left[P\right]}_{t}-{\left[L\right]}_{t}+{K}_{d}\left(1+{K}_{U}\right)\right)}^{2}+4{\left[L\right]}_{t}{K}_{d}(1+{K}_{U})} \right]$$

The term ${f}_{U}$ is the fraction of unfolded protein based on the concentration of unfolded protein, [U], divided by the total protein, [P]_t_, which is the sum of [U], folded protein [F], and folded protein with bound ligand [FL]. [L] is the total free ligand by correcting the total ligand concentration, [L]_t_, which is a main benefit of this model. K_U_ represents the equilibrium between folded and unfolded protein, and K_d_ is the ligand dissociation constant from the bound to unbound states.

3 $${K}_{d}={K}_{a}^{-1}=\frac{\left[F\right][L]}{[FL]}$$

### Hill adsorption model

The Hill model has been used to correct for the presence of a fluorescent protein–ligand complex and binding cooperativity^21,34^.

4 $$\frac{{I}_{0}-I}{{I}_{0}-{I}_{res}}=\left[\frac{{\left.{\left(K\right.}_{Hill}\left[Q\right]\right)}^{{n}_{Hill}}}{1+{\left.{\left(K\right.}_{Hill}\left[Q\right]\right)}^{{n}_{Hill}}}\right]$$

I_0_ and I are the fluorescence intensity of BSA in the absence and presence of the ligand and I_res_ accounts for residual fluorescence at infinite quencher concentration. K_Hill_ and n_Hill_ are the Hill binding constant and the cooperativity constant, respectively, and [Q] is the concentration of added quencher (PFAS or FA).

### Supplementary Information

Supplementary Information.

## Data Availability

All data will be made available free of charge upon email request to the corresponding author.
