# Supplementary material for: Differential scanning fluorimetry to assess PFAS binding to bovine serum albumin protein
Source: Sci Rep. 2024 Mar 18;14:6501. doi: 10.1038/s41598-024-57140-9 (PMC10948889; doi:10.1038/s41598-024-57140-9)
Supplement: Supplementary file 1 — Supplementary Information. [file 41598_2024_57140_MOESM1_ESM.docx]

SUPPORTING INFORMATION

Differential Scanning Fluorimetry to Assess PFAS Binding to Bovine Serum Albumin Protein

Jessica Alesio† and Geoffrey D. Bothun*,†

†Department of Chemical Engineering, University of Rhode Island, Kingston, Rhode Island 02881, United States.

*Corresponding author. **Tel:** +1-401-874-9518, **E-mail:** gbothun@uri.edu

Figure S1. An exemplary graph for perfluorononanoic acid (PFNA) showing the fraction of unfolded bovine serum albumin (BSA) protein, $f_{U}$, as a function of temperature for PFNA:BSA molar ratios from 0:1 to 4:1. The curves were generated from differentia scanning fluorimetry (DSF) data of tryptophan fluorescence intensity as a function of temperature. Values for $f_{U}$ are used in equations (1) and (2) to determine K_d_, which is equal to K_a_^-1^, at a given temperature.
